# Biomarker investigations related to pathophysiological pathways in schizophrenia and psychosis

**DOI:** 10.3389/fncel.2013.00095

**Published:** 2013-06-26

**Authors:** Gursharan Chana, Chad A. Bousman, Tammie T. Money, Andrew Gibbons, Piers Gillett, Brian Dean, Ian P. Everall

**Affiliations:** ^1^Department of Psychiatry, Melbourne Brain Centre, The University of MelbourneParkville, VIC, Australia; ^2^Department of Electrical and Electronic Engineering, Centre for Neural Engineering, The University of MelbourneParkville, VIC, Australia; ^3^Victoria Research Laboratory, Department of Electrical and Electronic Engineering, National Information and Communications Technology Australia, The University of MelbourneParkville, VIC, Australia; ^4^Florey Institute of Neuroscience and Mental HealthParkville, VIC, Australia; ^5^Molecular Psychiatry Laboratory, Florey Institute of Neuroscience and Mental HealthParkville, VIC, Australia

**Keywords:** schizophrenia, psychosis, gene expression regulation, biomarkers, postmortem brain

## Abstract

Post-mortem brain investigations of schizophrenia have generated swathes of data in the last few decades implicating candidate genes and protein. However, the relation of these findings to peripheral biomarker indicators and symptomatology remain to be elucidated. While biomarkers for disease do not have to be involved with underlying pathophysiology and may be largely indicative of diagnosis or prognosis, the ideal may be a biomarker that is involved in underlying disease processes and which is therefore more likely to change with progression of the illness as well as potentially being more responsive to treatment. One of the main difficulties in conducting biomarker investigations for major psychiatric disorders is the relative inconsistency in clinical diagnoses between disorders such as bipolar and schizophrenia. This has led some researchers to investigate biomarkers associated with core symptoms of these disorders, such as psychosis. The aim of this review is to evaluate the contribution of post-mortem brain investigations to elucidating the pathophysiology pathways involved in schizophrenia and psychosis, with an emphasis on major neurotransmitter systems that have been implicated. This data will then be compared to functional neuroimaging findings as well as findings from blood based gene expression investigations in schizophrenia in order to highlight the relative overlap in pathological processes between these different modalities used to elucidate pathogenesis of schizophrenia. In addition we will cover some recent and exciting findings demonstrating microRNA (miRNA) dysregulation in both the blood and the brain in patients with schizophrenia. These changes are pertinent to the topic due to their known role in post-transcriptional modification of gene expression with the potential to contribute or underlie gene expression changes observed in schizophrenia. Finally, we will discuss how post-mortem studies may aid future biomarker investigations.

## Introduction

The official national institute of health (NIH) definition of a biomarker is, “a characteristic that is objectively measured and evaluated as an indicator of normal biologic processes, pathogenic processes, or pharmacologic responses to a therapeutic intervention” (Nih, 2001). Investigations of blood-based biomarkers for schizophrenia have great potential in discovering diagnostic or prognostic indicators of clinical utility and may provide clues to the underlying pathophysiology as the blood is a dynamic, sentinel tissue that is known to reflect immune and pathological status (Gladkevich et al., 2004). Data from post-mortem investigations of gene and protein expression in schizophrenia can act two-fold in order to aid blood-based biomarker discovery. They may act to drive blood-based biomarker investigations by the genes, proteins, or structures that have been previously implicated, or conversely, as a means of extending findings seen in blood-based biomarker investigations. While biomarkers for disease do not need to have pathophysiological roles and can be utilized to detect presence or absence of disease, the ideal scenario maybe the identification of biomarkers that are involved in etiological pathways and are therefore likely to change according to disease status as well as potentially being responsive to treatments. Over the last two decades numerous post-mortem neuropathological investigations have implicated a number of different proteins in the pathogenesis of major psychiatric disorders, including schizophrenia, bipolar disorder, and major depressive disorder. Although some of these findings have been corroborated independently, many of them remain controversial and conflict with follow-up investigations. Similarly, gene association studies have been plagued with inconsistencies, with regards to genes implicated in individual populations falling out in meta-analyses or large independent replication efforts failing to validate initial findings. The advent of microarray technology, coupled with the mapping of the human genome, has greatly aided the ability to investigate larger numbers of genes simultaneously. While a number of these investigations have led to the generation of false-positive and -negative findings in terms of disease related gene expression changes some consistent trends are beginning to emerge.

In post-mortem gene expression investigations, alterations in the expression of transcripts encoding for oligodendrocyte functioning and myelination have been observed in the PFC in schizophrenia (Hakak et al., 2001; Tkachev et al., 2003, 2007; Aston et al., 2004, 2005; Kerns et al., 2010) and also in bipolar and major depressive disorder (Tkachev et al., 2003; Aston et al., 2005; Sun et al., 2006; Kim and Webster, 2011). These include genes encoding for major myelin components such as myelin basic protein (MBP) as well as myelin oligodendrocyte basic protein (MOBP). Such genes have been postulated to be involved in higher cognitive functions (Nave, 2010), functions which are compromised in patients with schizophrenia. While the functions of genes such as MOBP are not fully understood they are likely to have a significant role in the compaction of myelin sheaths, thereby aiding brain connectivity and signaling. Post-mortem schizophrenia investigations have also revealed decreased myelin thickness (Flynn et al., 2003) and damaged myelin sheaths (Uranova et al., 2011). In addition, recent diffusor tensor imaging (DTI) studies have demonstrated improvement in myelin integrity upon exposure to anti-psychotics in treatment responsive patients (Garver et al., 2008; Bartzokis et al., 2012), suggesting an alternative mechanism of action that may provide new targets for future development (Walterfang et al., 2011; Ren et al., 2013).

Genes related to metabolism and mitochondrial pathways are another ontological group that have been found by a number of studies to be reduced in expression in the PFC in schizophrenia (Pongrac et al., 2002; Tkachev et al., 2003; Prabakaran et al., 2004; Iwamoto et al., 2005; Konradi, 2005) and by some in bipolar disorder (Iwamoto et al., 2005). While the association of these genes with the disorders themselves may be evidenced, their mechanism of action in causing symptoms associated with the disorders remains to be elucidated. However, some studies have demonstrated that changes in gene expression are also a function of duration of illness which could be related to changing symptom profile that occurs with progression of illness (Narayan et al., 2008; Tang et al., 2009, 2012). Teasing out this etiological component is confounded by major psychiatric disorders having overlapping symptomatology, and in the case of schizophrenia and bipolar disorder sometimes leading to differential diagnosis. This problem, has prompted some researchers to look at key characteristic such as psychosis that provide a more tangible target to assess similarities and differences in etiology between disorders. This review will begin with an overview of the main post-mortem findings contributing to our understanding of the etiology and pathophysiology of schizophrenia and psychosis. Following this, current literature relating to blood-based gene expression studies in patients with schizophrenia and how they relate to post-mortem data will be discussed as well as the future direction of post-mortem investigations to validate blood-based biomarker findings. In relation to regulation of gene expression we will also discuss recent interesting findings of microRNA (miRNA) dysregulation in schizophrenia.

## Post-mortem data elucidating pathophysiology pathways in schizophrenia

### Dopaminergic system

Numerous post-mortem investigations in schizophrenia have implicated several neurotransmitter systems in having a pathophysiological role. The first of these centered on the dopaminergic system, primarily driven by the fact that neuroleptic medications had a high affinity for D2 receptors which correlated with their therapeutic effect at alleviating positive symptoms (Meltzer and Stahl, 1976). While this is supported by the observation that drugs such as amphetamine, cocaine and methamphetamine (METH) all increase dopaminergic transmission and are capable of inducing psychosis (Janowsky and Risch, 1979; Chen et al., 2003), other drugs such lysergic acid diethylamide (LSD), ketamine and phencyclidine (PCP) that also have the ability to produce psychosis do so without pronounced effects on the dopaminergic system. While early studies reported increased D2 receptor density in patients with schizophrenia vs. controls, these investigations did not control for the patients receiving neuroleptic medication and subsequent studies controlling for this confound (Reynolds et al., 1981; MacKay et al., 1982; Kornhuber et al., 1989) or having appropriately designed methodology (Dean et al., 1997) failed to detect differences. Post-mortem investigations have also found evidence for dysregulation in the gene for catechol-o-methyl transferase (*COMT*), encoding for the enzyme that degrades dopamine. These investigations have found that *COMT* gene expression is inversely correlated with gene expression of regulator of g-protein signalling-4 (*RGS4*) (Lipska et al., 2006), with substitutions at Val158Met in *COMT* significantly reducing the activity of COMT (Chen et al., 2004). These investigations are in keeping with a substantial gene association literature implicating polymorphism in COMT to schizophrenia susceptibility (Shifman et al., 2002; Tunbridge et al., 2006). However, as for many genes in the schizophrenia literature meta-analytic review of gene association investigations implicating *COMT* have shown that the findings of association do not hold up when comparing across studies (Glatt et al., 2003).

### Cholinergic system

The cholinergic muscarinic receptors (CHRM) have been implicated in the pathophysiology of schizophrenia as well as mood disorders, where psychosis can present as part of the symptomatology (Crook et al., 2000; Gibbons et al., 2009). In schizophrenia, post-mortem studies of the cortex point to the involvement of CHRM1, with the recent identification of a sub-type of schizophrenia with widespread loss of cortical and sub-cortical CHRM1 protein expression (Scarr et al., 2009; Gibbons et al., 2013). These post-mortem studies have been supported by a neuroimaging study demonstrating a reduction in muscarinic receptor availability in unmedicated patients with schizophrenia (Raedler et al., 2003) Whereas, post-mortem, neuroimaging, and genetic association studies implicate CHRM2 in the pathophysiology of mood disorders (Wang et al., 2004; Gibbons et al., 2009). Interestingly, elucidating the role of the cholinergic system in the etiology of psychosis has been aided by studies into Alzheimer's disease where the CHRM1/CHRM4 partial agonist Xanomeline can reduce psychotic symptoms (Bymaster et al., 1997; Shekhar et al., 2008). The cholinergic system has also been implicated in acute stress with inhibitors of the acetylcholine-hydrolyzing enzyme acetylcholinesterase (AChE) producing symptoms of depression and cognitive decline in mice via potentiation of acetylcholine signaling (Kaufer et al., 1998). In addition, a recent study in mice found that anxiety and depression behaviors elicited through administration of physostigmine, an inhibitor of AChE could be reversed with administration of nicotinic and muscarinic receptor antagonists (Mineur et al., 2013). These studies point to the potential of drugs that target the cholinergic system in alleviating some of the negative symptoms associated with schizophrenia, something that is poorly addressed by current antipsychotic medications.

Murine knockout models have shown that the antipsychotic effects of Xanomeline are predominantly mediated via CHRM4, which in turn modulates dopaminergic activity (Woolley et al., 2009). Post-mortem studies have also reported reduced binding of the radioligand [^3^H]4-DAMP, under CHRM1/CHRM3/CHRM4/CHRM5 selective assay conditions, in the orbitofrontal cortex from subjects with Alzheimer's disease who had significant psychotic symptoms (Tsang et al., 2008). Conversely, elevated levels of the cortical autoreceptor CHRM2 (Zhang et al., 2002) are associated with behavioral disturbances in both Alzheimer's disease and Lewy body dementia (Lai et al., 2001; Teaktong et al., 2005) pointing to a hypocholinergic state associated with psychosis. In schizophrenia, subjects with deficits in CHRM1 protein expression also show a reduction in cortical and subcortical binding of [^3^H]AF-DX 384, a CHRM2/CHRM4 selective radioligand (Gibbons et al., 2013). The reduction in hippocampal CHRM4 mRNA levels in schizophrenia suggests this loss of binding reflects a loss of CHRM4 protein expression (Scarr et al., 2007). Within the cortex, [^3^H]AF-DX 384 binding is reported to be unchanged in the ACC in schizophrenia. However, there is contrasting data relating to the role of CHRM4 in schizophrenia (Crook et al., 1999; Zavitsanou et al., 2005; Gibbons et al., 2013). Thus, cholinergic involvement in psychosis may be specific to a sub-type within schizophrenia. Importantly, a SPECT study has reported reduced binding densities of the broadly selective CHRM radioligand [^123^I]IQNB in patients with schizophrenia (Raedler et al., 2003) raising the possibility of clinical identification of individuals within this subtype using neuroimaging.

### Glutamatergic system

A role for glutamate, the primary excitatory neurotransmitter in the brain, has long been evidenced from observations that drugs such as ketamine and phencyclidine (PCP) that primarily block iontropic n-methyl-d-aspartate receptors (NMDARs) and are capable of causing negative and positive symptoms that resemble those seen in psychotic disorders such as schizophrenia (Luby et al., 1962; Vollenweider and Geyer, 2001; Pomarol-Clotet et al., 2006). This observation led to the NMDA hypofunction hypothesis of schizophrenia (Olney and Farber, 1995) with the involvement of the glutamatergic system in psychosis stemming from neuroimaging, genetic, and post-mortem investigations. In support of a glutamatergic dysfunction in schizophrenia, kainate receptor expression is decreased in the dlPFC in schizophrenia (Scarr et al., 2005), while hippocampal expression of the NR1 and NR2B subunits of the NMDA receptor are increased and decreased, respectively (Gao et al., 2000). Other studies have shown that NMDARs are likely involved with schizophrenia including early studies demonstrating that glutamate carboxypeptidase II (GCPII) that catabolizes N-Acetylaspartylglutamic acid (NAAG) to NAA and glutamate was reduced in the frontal cortex, hippocampus and temporal lobe in patients with schizophrenia vs. controls (Tsai et al., 1995) as well as more recent evidence demonstrating that GAD67 neurons co-expressing NR2A are reduced in the prefrontal cortex in patients with schizophrenia (Woo et al., 2004, 2008). Increased mRNA and protein levels of the glutamate transporters EAAT1, EAAT3, and EAAT4 have also been reported in schizophrenia (Rao et al., 2012). Furthermore, many of the genes that have consistently been associated with schizophrenia, such as *NRG1*, *RGS4*, *DTNBP1*, and *DAAO* are involved in regulation of glutamatergic neurotransmission, with the antipsychotic clozapine being shown to increase levels of *NRG1* as well as vesicle-associated membrane protein-1 *(VAMP1)* in human primary cultures exposed at steady state plasma levels (Chana et al., 2009), indicating that *NRG1* may be a possible therapeutic target. Indeed NRG1 signaling through ErbB4 receptors have the capacity to determine NMDA efficacy, with NMDA receptor activity playing a key role in the development and stability of synaptic spines (Bennett, 2009), with stress responses and activation of the glucocorticoid receptor system also causing synaptic regression (Bennett, 2008).

With regards to modulation of NMDA receptors and the NMDA hypofunctioning hypothesis of schizophrenia, DAAO is of particular interest due to its metabolism of d-serine, a known allosteric modulator and necessary co-agonist for NMDA receptors (Oliet and Mothet, 2009). DAAO levels have been found to be elevated in both the brain and cerebrospinal fluid (CSF) of patients with schizophrenia (Bendikov et al., 2007; Madeira et al., 2008; Habl et al., 2009). This in turn results in a reduction in the breakdown of d-serine and could facilitate NMDA hypofunctioning. In relation to psychosis, *DAOA* has recently been linked to progression to a first episode of psychosis in prodromal subjects (Mossner et al., 2010). Another two important proteins that support such a hypothesis are d-serine racemase, a predominantly astrocytic enzyme that converts l-serine to d-, and d-amino-acid oxidase activator (DAOA) that as it name suggests may be responsible for the activation of DAAO and hence subsequent breakdown of d-serine. However, while one would expect that serine racemase would be reduced in a scenario of NMDA hypofunctioning i.e., reduced d-serine production and therefore reduced NMDA activation, increased serine racemase has been reported within the frontal cortex in a post-mortem study of schizophrenic subjects (Verrall et al., 2007) as well as decreased (Bendikov et al., 2007). In addition, an earlier study reported no change in the cortex but an increase in expression within the hippocampus (Steffek et al., 2006). Therefore, as a correlate for NMDA hypofunctioning further characterization is required in independent brain cohorts before a firm conclusion can be reached. The evidence for DAOAs involvement in psychosis has been growing with several genetic studies finding an association between polymorphisms in the *G72* gene which encodes DAOA, and the development of psychosis (Addington et al., 2004; Craddock et al., 2005; Mossner et al., 2010) as well as severity of positive symptoms (Chiesa et al., 2011). In addition, a recent investigation demonstrated that polymorphisms within the *G72* gene were associated with a vulnerability to methamphetamine psychosis (Kotaka et al., 2009). While the data from the study may seem damning to aspirations of DAOA as a susceptibility gene for psychosis, the authors for this study did not differentiate between brain regions other than the amygdala and the caudate nucleus. Therefore, it was unclear whether key brain regions such as the PFC were included and in what proportions. Furthermore, there is also a need for separation of cortical and subcortical matter due to potential differences in expression that may exist between them.

### GABAergic system

Gamma-amino-butyric-acid (GABA) has been implicated for a number of years in schizophrenia following a number of post-mortem investigations demonstrating its dysregulation within the brain. The most consistent of these changes and indeed one of the most consistent post-mortem neuropathological changes observed for schizophrenia were reductions in GABAergic markers such as glutamic acid decarboxylase-67 (GAD-67), the synthesizing enzyme for GABA, within the PFC (Akbarian et al., 1995; Guidotti et al., 2000; Volk et al., 2000; Veldic et al., 2005; Akbarian and Huang, 2006) as well as the temporal cortex (Impagnatiello et al., 1998; Heckers et al., 2002; Akbarian and Huang, 2006) and hippocampus (Thompson et al., 2011). In relation to these studies, and as a potential link for GABA's involvement in psychosis, Veldic et al. (2005) demonstrated a decreased number of neurons expressing mRNA for GAD-67 within the PFC of brains from schizophrenia and bipolar subjects with a history of psychosis with a concomitant increase in cortical DNA-methyltransferase I (DNMT1), an enzyme preferentially expressed by interneurons, causing a possible down-regulation of promoter functioning within these cells (Veldic et al., 2005). Reductions in GABAergic functioning have also been evidenced by reduced expression of a number of the subunits making up the GABAA receptor by (Charych et al., 2009). Furthermore, while GAD-65 and GAD-67 mRNA expression has been shown to be either reduced or unchanged within the layers of the hippocampus in subjects with schizophrenia (Benes et al., 2007), increases in GAD-65/67 immunoreactive neuronal density has been reported in the subiculum and parahippocampal gyrus from chronically medicated cases (Schreiber et al., 2011); suggesting that increasing GABA synthesis may be a function of antipsychotics.

### Serotoninergic system

While blockade of D2 receptors would seem to be a requirement for both typical and atypical antipsychotic medications in alleviating positive symptoms, atypicals such as clozapine are also known to have a high affinity for serotonergic receptors, in particular 5HT_2A_ receptors (2ARs) (Meltzer, 1992). This observation has led some researchers to elucidate a possible role for serotonin in both the positive and negative aspects of schizophrenia. While many post-mortem studies have demonstrated decreases in 2ARs (Burnet et al., 1996; Dean et al., 1996; Kouzmenko et al., 1997) a recent study by Gonzalez-Maeso et al. (2008) demonstrated an upregulation of 2ARs, and a concomitant decrease in mGLUR2 within the PFC of untreated patients who had schizophrenia. (Gonzalez-Maeso et al., 2008). One important aspect of this study that distinguishes it from previous post-mortem investigations of 2AR was that the brains of their subjects who had schizophrenia were anti-psychotic naïve and therefore changes in expression could not be explained medication effects. Indeed in order to better understand the discrepancy between their findings and others the authors investigated 2AR expression changes associated with treating mice with the atypical antipsychotic clozapine and typical antipsychotic haloperidol. They found that clozapine downregulated 2AR expression in the somatosensory cortex while this effect was not seen for haloperidol. While the potential cross-talk between mGLUR2 and 2AR may have been demonstrated within this investigation and is of interest, a major oversight by the authors was in not commenting on the impact of a high number of suicide victims in their schizophrenia cohort, with 87.5% of all cases dying by this mode, compared to a rate of 4.9% estimated in a recent re-examination of rates within the schizophrenia population (Palmer et al., 2005). This is of importance given that 2ARs have been demonstrated to be elevated within the frontal cortex of victims of suicide (Stanley and Mann, 1983; Arranz et al., 1994; Turecki et al., 1999), which in turn could potentially explain the elevated 2ARs seen in their study. However, the proposed role for 2AR in psychotic illness is concordant with findings in Parkinson's disease where binding the 2AR radioligand [^3^H] ketanseron is increased in cases with a history of visual hallucinations (Huot et al., 2010). While evidence has been mounting for the independent contributions of each of the aforementioned neurotransmitter systems to the development of psychosis the co-ordination or dysregulation between neurotransmitters is a more logical likely causation of psychosis. This interactive approach when viewing neurochemical abnormalities in schizophrenia and psychosis has come about in part due to our understanding of individual neurotransmitter systems and how they intermingle to regulate the action of one another. It has also come about following a realization that psychosis-related disorders are too complex for their pathophysiology to be underpinned by a single neurotransmitter.

## Functional neuro-imaging data elucidating pathophysiology pathways in schizophrenia

Over the last decade the power of functional neuroimaging studies to characterize pathophysiological pathways in schizophrenia has greatly increased, especially in the advancement of studies using positron emission tomography (PET) and single-photon emission computed tomography (SPECT) that are techniques allowing the visualization in real-time of changes in receptor densities related to neurotransmitter and signaling systems. Importantly, this has allowed the validation of post-mortem findings as well as expansion of implicated pathways for schizophrenia.

### Dopaminergic system

Probably the most well-investigated neurotransmitter system using these imaging modalities has been the dopaminergic system. As a main molecular target for antipsychotic medications, dopamine, and D2 receptor binding has been studied by a number of investigations utilizing nuclear magnetic spectroscopy (NMS) as well as PET and SPECT, with the caudate nucleus being the brain area most heavily investigated due to it's high D2 receptor density. While some investigations utilizing NMS have reported increased binding in drug naïve and late onset schizophrenia patients (Tune et al., 1993, 1996; Pearlson et al., 1995) others have failed to detect such changes (Hietala et al., 1994; Nordstrom et al., 1995). This lack of congruence amongst studies may represent differing methodologies and pharmacology of radioligands used (Dean, 2012), but may also be indicative of a similar lack of consistency for D2 receptor density and occupancy in post-mortem brain tissue. Potential confounders of these investigations are age and duration of illness, with post-mortem studies showing that D2 occupancy decreases with age (Rinne et al., 1993; Dean et al., 1997) and more recent evidence demonstrating that gene expression of D2 changes according to increasing duration of illness (Dean et al., 2007). Similar controversy has surrounded investigations in schizophrenia that assessed levels of dopamine-D1 like receptors, with decreases reported in a number of different brain regions (Okubo et al., 1997; Kosaka et al., 2010) in contrast shown to be increased by others (Abi-Dargham et al., 2012). In addition, post-mortem studies also reflect variability in findings related to D1 receptor levels (Seeman et al., 1987; Knable et al., 1994; Dean, 2012). A major confounding variable of these studies is likely related to the variation in medication histories between individuals. As for dopamine receptors, PET and SPECT studies assessing levels of dopamine transporter (DAT) have also produced inconsistency in findings, with some investigations reporting decreased DAT levels (Laakso et al., 2001; Mateos et al., 2007) but with the majority of investigations reporting no differences (Kuepper et al., 2012). Therefore, the change in DAT levels, if present in schizophrenia, are likely to be of a small magnitude. While dopamine receptor and transporter studies have failed to show consistent changes in patients with schizophrenia, investigations aimed at assessing dopamine synthesis and release have provided evidence for neurotransmitter changes in the striatum. Using [18F]flurodopamine a number of studies have demonstrated increased uptake of this radioligand in the striatum in patients with schizophrenia (Hietala et al., 1995, 1999; Laruelle et al., 1996). These findings are also consistent with amphetamine-induced dopamine release being significantly increased in the striatum of patients with schizophrenia using the ligand [123]iodobenzamide (Laruelle et al., 1996) and while it could be argued that these changes may be related to medication effects, increased dopamine release has also been observed in drug-naïve and drug free patients (Abi-Dargham et al., 1998, 2009). These findings warrant further investigation in additional cohorts.

### Cholinergic system

While post-mortem investigations of the cholinergic system have revealed evidence for decreases in M1 receptors in schizophrenia (Scarr et al., 2009; Gibbons et al., 2013), functional neuroimaging investigations of the cholinergic system have been less numerous. Nevertheless, a study using the ligand [^123^]iodoquinclidinyl benzilate ([123I]IQNB) reported widespread reductions in muscarinic receptors in the brains of drug free schizophrenia patients(Raedler et al., 2003). Given that reductions in muscarinic receptors have also been observed in bipolar disorder and major depressive disorder (Gibbons et al., 2009) the investigation of the muscarinic system as a common system affected in major psychiatric disorders may be warranted.

### Glutamatergic system

With ketamine and PCP capable of causing psychosis through NMDA receptor blockade the limited number of functional imaging studies for glutamate in schizophrenia have focused on characterizing potential changes in NMDA signaling. These studies have shown that there is no change in the thalamus and striatum using the ligand [^123^] CNS 1261 (Bressan et al., 2003), with the same group showing that there was a decrease in NMDA receptors in the left hippocampus (Pilowsky et al., 2006). With growing evidence that the glutamatergic system plays an important role in the etiology of schizophrenia, including the role of metabotropic glutamate receptors and their cross-talk with NMDA receptors, further functional neuroimaging investigations generating ligands for other glutamatergic targets is a likely outcome within the next decade.

### GABAergic system

While GABAergic dysfunction in schizophrenia has been elucidated by a number of post-mortem investigations demonstrating alterations in interneuronal populations in the frontal and cingulate cortices, imaging studies have been limited with regards to assessing functional changes in GABA transmission. Investigations that have looked at GABA_A_ receptors have found that there are no changes in levels of this receptor in the brain in patients with schizophrenia (Busatto et al., 1997; Abi-Dargham et al., 1999; Verhoeff et al., 1999). In addition, a PET investigation that investigated levels of the central benzodiazepine receptor (cBZr) located on the GABA_A_ receptor in patients with anxiety and controls found no differences (Abadie et al., 1999).

### Serotonergic system

The focus of functional neuroimaging studies in relations to the serotonergic system has been on the 5HT2AR receptors, due to clozapine, as the first of the second generation antipsychotic medications demonstrating a high affinity at 2ARs (Meltzer, 1992). Interestingly, as for the post-mortem studies, decreased 2ARs have also been observed by PET studies (Ngan et al., 2000; Rasmussen et al., 2010), with the latter of these studies being carried out in drug-naïve subjects. Decreased 2AR density has additionally also been seen in individuals at risk of developing mental illness (Hurlemann et al., 2008). Contrary to these investigations, however, a study conducted in first-episode neuroleptic naïve subjects found a 40% increase in 2ARs (Erritzoe et al., 2008). This controversy in functional neuroimaging studies mirrors that for post-mortem brain tissue and also reflects findings above for other neurotransmitter systems. With an increase in superior ligands for neurotransmitter receptors in the coming years, a consensus on *in vivo* receptor changes and how they relate to post-mortem findings will be important in order for us to better understand longitudinal changes in these receptor populations according to disease.

## Blood based gene expression investigations in schizophrenia

For psychotic and brain disorders the CSF represents the most obvious choice as a tissue source for detecting biomarkers in living individuals. However, as obtaining CSF samples is a substantially more invasive procedure than simply drawing blood, a rapidly growing number of investigations have focused on the use of blood to search for biological indicators of psychosis and schizophrenia. Early blood based biomarker investigations found evidence for alterations in neurotransmitter expression within blood platelets from patients with schizophrenia. These studies included a positive correlation between dopamine uptake from platelets from patients with schizophrenia and delusional state (Tateno et al., 2013) as well as increases in platelet 5HT-2 receptors that is increased further following treatment with fluphenazine and trifluoperazine (Amato et al., 2011). A more recent study has demonstrated that loxapine treatment downregulates both D2-like and 5HT-2A in platelets from patients with schizophrenia (Nord and Farde, 2011), with the latter finding being contradictory to that by Pandey et al. Further evidence indicating that platelets of patients with schizophrenia may provide a suitable tissue for a source of biomarker discovery has come from another recent study demonstrating that platelets derived from patients with schizophrenia demonstrated hypoglutamatergic release of Ca^2+^ (Miyake et al., 2011). Further characterization work is needed in order to uncover what other neurotransmitter receptors these cells express.

A number of recent blood-based expression studies have utilized lymphoblastoid cells cultured from patients with schizophrenia and bipolar disorder. The earliest one of these studies was that by Kakiuchi et al. (2003) in which a reduction in expression of X-box binding protein 1 (*XBP1*) in a pair of twins discordant for bipolar disorder vs. a control twin pair was reported. XBP1 is a pivotal gene in the endoplasmic reticulum (ER) stress response and therefore is of interest given elevation of stress response molecules such as cortisol in patients with bipolar disorder. In addition, they found that a polymorphism substitution at position 116 (C—G) in the promoter region of *XBP1* lymphoblasts derived from Japanese patients with bipolar disorder conferred a reduced ER stress response that was rescued by treatment with the mood stabilizer valproate (Kakiuchi et al., 2003). More recently, they extended their findings by demonstrating that lithium treatment also was more effective in patients with the 116C allele as opposed to patients homozygous for 116G (Kakiuchi et al., 2006). While this finding directly relates the codon substitution to a known mood-stabilizing agent, validation of this interesting finding in a separate larger independent patient cohort is warranted. In the largest microarray study of lymphoblastoid cells so far by Vawter et al. (2004) it was found that the expression of neuropeptide Y (*NPY*) and the gene that encodes for Guanine nucleotide-binding protein G(o) subunit alpha (*GNA01)* were reduced in schizophrenia and there was an increase in the mitochondrial-related gene malate dehydrogenase 1, NAD (*MDH1*) (Vawter et al., 2004). Changes in NPY have been observed by some post-mortem investigations in schizophrenia and bipolar disorder and are therefore of interest (Frederiksen et al., 1991; Iritani et al., 2000; Kuromitsu et al., 2001). It should be noted that even for this study sample numbers were relatively small with only 5 patients with schizophrenia and 9 controls. Moreover, studies that used lymphoblastoid cell lines *utilized* Epstein Barr virus (EBV) to transform PBMCs, an effect which in itself could lead to changes in gene expression.

Peripheral blood microarray findings have implicated sensory and motor neuron derived factor (*SMDF*), a splice variant of *NRG1*, in patients with schizophrenia (Petryshen et al., 2005). In addition, decreased gene expression of *NRG1* and dystrobrevin binding protein 1 (*DTNBP1*) were shown to be lower in immortalized lymphocytes from patients with schizophrenia vs. controls before and after treatment with the antipsychotic olanzapine (Chagnon et al., 2008). However, a follow-up study failed to report changes in *NRG1*, *DTNBP1*, or *GNAO1* in patients with schizophrenia vs. controls (Yamamori et al., 2011). Reduced expression of *NRG1* has also been observed in PBMCs from first-onset schizophrenia patients compared to controls, which normalized following treatment with antipsychotic medications (Zhang et al., 2008). Interestingly, another microarray gene expression investigation using whole blood from patients with schizophrenia and assessing gene expression changes related to positive symptomatology, found that *NRG1* was increased relative to higher delusional states (Kurian et al., 2011). This contradictory finding may be a consequence of a lack of control group for the latter investigation or may be related to the separation of symptomatology that was carried out. This finding for *NRG1* reflects the genetic association studies that have found a susceptibility for schizophrenia as well as recent evidence for reduced *NRG1* levels in the PFC of brains of patients with schizophrenia and unipolar depression (Bertram et al., 2007). While some studies have failed to demonstrate association of *NRG1* to schizophrenia, (Thiselton et al., 2004; Duan et al., 2005; Rosa et al., 2007) recent meta-analyses have supported a relationship (Li et al., 2006; Munafo et al., 2006). Impaired *NRG1* migration of B-lymphocytes isolated from patients with schizophrenia vs. controls has also been demonstrated that was associated with polymorphisms in *NRG1* and the gene for catechol-o-methyl-transferase [*COMT* (Sei et al., 2007)], an enzyme required for degradation of neurotransmitters including dopamine and that has also been heavily implicated in schizophrenia via association studies (Williams et al., 2007). However, it is important to mention that findings for *COMT* have been relatively inconsistent and a recent meta-analysis failed to find an association with *COMT* and susceptibility to schizophrenia (Okochi et al., 2009). Of relation to biomarkers of dopaminergic signaling, another investigation also demonstrated that the number of leukocytes expressing dopamine and cyclic adenosine 3″:5″-monophosphate regulated phosphoprotein of relative molecular mass 32,000 (DARPP-32) was reduced within PBMC sub-populations in schizophrenia and bipolar disorder (Williams et al., 2007; Torres et al., 2009).

In a comparative blood and brain gene expression study we demonstrated that the gene for selenium binding protein-1 (*SELENBP1*) was upregulated in both compartments in schizophrenia and was validated in the blood using qRT-PCR and immunohistochemistry (Glatt et al., 2005). In a follow-up qRT-PCR study looking at a separate brain cohort, our group also observed increased *SELENBP1* expression in the dlPFC of patients with schizophrenia (Kanazawa et al., 2008). Interestingly, the expression of *SELENBP1* was significantly correlated with psychosis in a combined sample of schizophrenia and bipolar disorder patients regardless of their diagnosis. In contrast to these findings, a study of 30 patients first hospitalized for a psychotic episode with symptoms consistent with schizophrenia (*n* = 15), schizophreniform disorder (*n* = 13) or schizoaffective disorder (*n* = 2) investigated levels of *SELENBP1* in PBCs after commencement of treatment. Using qRT-PCR, *SELENBP1* was compared between patients with a psychotic disorder and controls; there was no significant difference between the groups (Yao et al., 2008). Nevertheless, a recent study investigated the expression of *SELENBP1* in BA46 from 13 subjects with major depressive disorder (MDD), 11 subjects with MDD and psychotic features and 12 controls (Teyssier et al., 2011). There was no significant difference in the expression of *SELENBP1* between subjects with psychotic features and controls, or subjects with a depressive disorder and controls (Teyssier et al., 2011). The authors argued that results demonstrate that *SELENBP1* expression is not a marker of psychosis; however, the sample size was small and may not have sufficient power to detect a difference in the expression of *SELENBP1*. *SELENBP1* is localized to chromosome 1q21, which has previously been identified as a susceptibility locus for schizophrenia (Brzustowicz et al., 2000), which indicates that the upregulation of *SELENBP1* in psychotic disorders may be involved in the susceptibility to psychosis. While increased expression of *SELENBP1* in the dlPFC in schizophrenia may be present, the functional relevance of this upregulation remains to be elucidated. One potential mechanism may be related to its potential role as a neurogenic factor evidenced by two investigations demonstrating its ability to promote neurite outgrowth; in the rat cerebral cortex (Zhao et al., 2000) as well as co-localization with actin in growing tips of human neuroblastoma SH-SY5Y cells (Miyaguchi, 2004). Given that reductions in synaptic and dendritic arbors have been observed in the brains of patients with schizophrenia, elevated *SELENBP1* may play a compensatory role in restoring neuronal connectivity and functioning. Further work to better define this tentative mechanism is being carried out. SELENBP1 has also been implicated in ubiquitination from a study demonstrating the binding of SELENBP1 to von Hippel- Lindau protein (pVHL)- interacting deubiquitinating enzyme 1 (VDU1) that was abolished with the incubation of β-mercaptoethanol, which dissociates selenium (Jeong et al., 2009), suggesting a selenium dependent interaction of SELENBP1 and VDU1.

Another recent blood based finding has been a reported increase in lipid biosynthesis in schizophrenia patients treated with olanzapine vs. untreated patients (Vik-Mo et al., 2008). In particular the authors found an increase in gene expression of fatty acid synthase (FASN) and stearoyl-CoA desaturase (SCD) potentially implicating these genes in adverse side effects such as weight gain. Calcineurin (CaN) has been shown to be down regulated within the hippocampus of subjects with schizophrenia (Eastwood et al., 2005) and has also been found to be correlated with psychopathology as measured by the brief psychiatric rating scale (BPRS) (Murata et al., 2008). Another blood-based biomarker investigation with a linkage to the brain was carried out by Hattori et al., who demonstrated that in platelets from patients with schizophrenia gene expression of Fyn, a member of the Src-kinase family which targets NMDA receptors for phosphorylation was increased (Hattori et al., 2009). In a microarray study looking at gene expression changes in leukocytes of both patients with schizophrenia and bipolar disorder vs. controls we identified dysregulation of the ubiquitin proteasome system (UPS) using pathway analysis (Bousman et al., 2010a). Follow-up examination of individual genes within the UPS showed ubiquitin conjugating enzyme 2 k (UBE2K) and seven in absentia homolog-2 (SIAH2) had positive correlations with positive symptom severity (Bousman et al., 2010b). These findings support previous schizophrenia post-mortem studies that have reported dsyregulated expression of several ubiquitin-related genes (Middleton et al., 2002; Altar et al., 2005). A role for the involvement of GSK3β signaling in schizophrenia has come from a number of studies including the demonstration that decreased AKT1 expression was present in the brain and lymphocytes of patients with schizophrenia (Emamian et al., 2004) that was recently replicated for lymphocytes at the gene expression level (van Beveren et al., 2012). Given GSK3β 's role in neural development, neurogenesis, neuronal differentiation as well as synaptic plasticity and axonal growth (Castano et al., 2010; Hur and Zhou, 2010) and that Akt1 phoshporylates GSK3β to inactivate these findings within the blood may reflect mechanisms taking place in the brain. These roles are of relevance to schizophrenia as reductions in synaptic and dendritic markers are amongst the most consistent post-mortem brain observations for the disorder (Jarskog et al., 2007). Lastly, there is growing evidence that there may be immune dysregulation in patients with schizophrenia and recent gene expression investigations within the blood have demonstrated changes in immune pathways in patients with schizophrenia (Gardiner et al., 2013; Xu et al., 2012). Table 1 below summarizes gene expression of biomarkers for schizophrenia that overlap in the blood and brain.

**Table 1:** **Gene expression biomarkers for schizophrenia in blood and brain**.

		**Tissue**		**Sample size**			**Gene expression**	
**Gene**	**Platform**	**Category**	**Type**	**N**	**SZ**	**Controls**	**Relative to controls**	**References**
CHRNA7	qRT-PCR	Blood	PBMC	55	34	21	↓ TNS & SZ	Perl et al., 2003
	qRT-PCR	Blood	PBMC	60	44	16	↓ SZ	Perl et al., 2006
	qRT-PCR	Brain	DLPFC	91	30	61	× SZ	
	qRT-PCR	Brain	HIP	91	30	61	× SZ	Mathew et al., 2007
DARPP-32	qRT-PCR	Blood	PBMC	15	8	7	↓ SZ	Torres et al., 2009
	qRT-PCR	Brain	DLPFC (BA46)	70	35	35	↓ SZ-S	Feldcamp et al., 2008
	qRT-PCR	Brain	PFC	67	33	34	↑ SZ	Zhan et al., 2011
	qRT-PCR	Brain	DLPFC	502	176	326	↑ SZ	Kunii et al., 2013
DISC1	qRT-PCR	Blood	PBMC	22	10	11	↑ TNS & SZ	Kumarasinghe et al., 2013
	qRT-PCR	Brain	HIP	91	30	61	↑ SZ	Nakata et al., 2009
DRD2	Custom	Blood	PBL	23	10	13	↑ TNS	Zvara et al., 2005
	qRT-PCR	Blood	PBMC	56	30	26	× SZ	Yao et al., 2008
	Affy U133 plus 2.0	Blood	PBMC	21	9	12	↑ SZ	Glatt et al., 2011
	qRT-PCR	Blood	PBMC	22	10	11	× TNS & SZ	Kumarasinghe et al., 2013
	qRT-PCR	Brain	PFC	67	33	34	× SZ	Zhan et al., 2011
DTNBP1	Agilent 18K	Blood	LCL	24	12	12	↓ SZ	Chagnon et al., 2008
	qRT-PCR	Blood	LCL	90	45	45	× SZ	Yamamori et al., 2011
	QISH	Brain	DLPFC	29	14	15	↓ SZ	Weickert et al., 2004
ERRB3	qRT-PCR	Blood	LCL	59	25	34	↓ SZ	Law et al., 2012
	Affy HuGeneFL	Brain	DLPFC	24	12	12	↓ SZ	Hakak et al., 2001
	Affy HgU95A	Brain	MTG (BA21)	26	12	14	↓ SZ	Aston et al., 2004
	Affy U133A&B	Brain	BA24/32, HIP	26	113	13	↓ SZ	Katsel et al., 2005
	qRT-PCR	Brain	DLPFC	103	31	72	↓ SZ	Law et al., 2012
ERRB4	qRT-PCR	Blood	LCL	59	25	34	↑ SZ	
	qRT-PCR	Brain	DLPFC	103	31	72	× SZ	Law et al., 2012
MAL	Affy HuGeneFL	Brain	DLPFC (BA46)	24	12	12	↓ SZ	Hakak et al., 2001
	qRT-PCR	Blood	PBMC	22	10	11	↓ TNS; × SZ	Kumarasinghe et al., 2013
MBP	qRT-PCR	Blood	PBMC	84	39	45	× TNS & SZ	Gutierrez-Fernandez et al., 2010
	Affy U133 plus 2.0	Blood	PBMC	21	9	12	↓ SZ	Glatt et al., 2011
	qRT-PCR	Blood	PBMC	22	10	11	↑ TNS; × SZ	Kumarasinghe et al., 2013
	qRT-PCR	Brain	PVC (BA17)	30	15	15	↓ SZ	Matthews et al., 2012
MDH1	Custom	Blood	LCL	14	5	9	↑ SZ	Vawter et al., 2004
	Affy HuGeneFL	Brain	DLPFC (BA46)	24	12	12	↑ SZ	Hakak et al., 2001
	UniGEM V2	Brain	PFC (BA9)	20	10	10	↓ SZ	Middleton et al., 2002
	qRT-PCR	Brain	DLPFC	43	22	21	↓ SZ	Vawter et al., 2004
NPY1R	Custom	Blood	LCL	14	5	9	↑ SZ	Vawter et al., 2004
	qRT-PCR	Blood	LCL	90	45	45	× SZ	Yamamori et al., 2011
	Affy HuGeneFL	Brain	PFC (BA10)	30	15	15	↓ SZ	Kuromitsu et al., 2001
	qRT-PCR	Brain	DLPFC	164	81	82	↓ SZ	Choi et al., 2008
NRG1	Affy U133 plus 2.0	Blood	PBL	38	33	5	↑ SZ	Petryshen et al., 2005
	Affy U133 plus 2.0	Blood	PBL	78	31	47	↑ FEP	Zhang et al., 2008
	Agilent 18K	Blood	LCL	24	12	12	↓ SZ	Chagnon et al., 2008
	qRT-PCR	Blood	LCL	90	45	45	× SZ	Yamamori et al., 2011
	qRT-PCR	Blood	PBL	163	80	83	↓ TNS; × SZ	Zhang et al., 2008
	qRT-PCR	Brain	DLPFC	39	20	19	↑ SZ^a^	Hashimoto et al., 2004
	qRT-PCR	Brain	HIP	91	38	53	↑ SZ^a^	Law et al., 2006
	qRT-PCR	Brain	PFC (BA10)	19	11	8	↓ SZ^a^	
	qRT-PCR	Brain	PFC (BA10)	19	11	8	↑ SZ^b^	
	qRT-PCR	Brain	PFC (BA9)	19	11	8	× SZ	
	qRT-PCR	Brain	HIP	19	11	8	× SZ	Parlapani et al., 2010
	qRT-PCR	Brain	PFC (BA9)	12	6	6	↑ SZ	
	qRT-PCR	Brain	HIP	11	6	5	× SZ	Marballi et al., 2012
PICK1	Affy U133 plus 2.0	Blood	PBMC	21	9	12	↑ SZ	Glatt et al., 2011
	qRT-PCR	Brain	DLPFC	70	35	35	↑ SZ	Sarras et al., 2010
PIK3CD	qRT-PCR	Blood	LCL	59	25	34	↑ SZ	
	qRT-PCR	Brain	DLPFC	103	31	72	× SZ	Law et al., 2012
PIK3R3	qRT-PCR	Blood	LCL	59	25	34	↑ SZ	
	qRT-PCR	Brain	DLPFC	103	31	72	↑ SZ	Law et al., 2012
SELENBP1	qRT-PCR	Blood	PBMC	58	34	24	↑ SZ	Glatt et al., 2005
	qRT-PCR	Blood	PBMC	56	30	26	× SZ	Yao et al., 2008
	Affy U133A	Brain	DLPFC	46	19	27	↑ SZ	Yao et al., 2008
	qRT-PCR	Brain	DLPFC	68	34	34	↑ SZ	Kanazawa et al., 2008

*BA, Brodmann area; DLPFC, dorsolateral prefrontal cortex; FEP, first episode psychosis; HIP, hippocampus; LCL, lymphblastoid cell line; MTG, middle temporal gyrus; PAC, parietal cortex; PBL, peripheral blood lymphocyte; PBMC, peripheral blood mononuclear cell; PFC, prefrontal cortex; PVC, primary visual cortex; QISH, quantitative in situ hybridization; SZ, schizophrenia; SZ-S, schizophrenia suicide; TNS, treatment niave schizophrenia*.

a *NRG1 Type I only*.

b *NRG1 Type II only*.

While blood based gene expression investigation offer great potential in identifying biomarkers for schizophrenia and psychosis, which could then ultimately be used as diagnostic and prognostic indicators the discovery of such markers may take some time to achieve. For blood-based gene expression studies there are several parameters that can substantially affect gene expression including diet, genetics, time to last meal, time of day, any medications used, and frequency of exercise (Radich et al., 2004). As well as these factors, technology such as microarrays while having the utility of assessing global gene expression changes, present problems in terms of consistency across studies and laboratorys. Indeed, a number of studies to date, looking at cross platform comparisons have unfortunately seen a very low level of correlation in gene expression (Tan et al., 2003; Hollingshead et al., 2005), even with mRNA samples derived from homogenous tissue sources such as cell lines. This has led to the conclusion that gene expression data cannot be combined reliably between platforms. Knowledge of this result has often led researchers to choose platforms on the basis of what past investigations have utilized. While the logic behind this choice is concrete, it does to a certain extent preclude an informed decision based on merits of individual platforms. In addition, many gene expression findings that are discovered through microarray investigations are often not validated by independent and more sensitive techniques such quantitative real-time PCR (qRT-PCR). Nevertheless, with the advent of more sensitive and comprehensive ways to analyse gene expression through next generation sequencing, the potential of blood based biomarker investigations may be realized sooner than expected.

## MicroRNAs and dysregulation in schizophrenia

Current evidence suggests that psychiatric illnesses are not the result of a dysfunctional single monoamine system but the disruption of an entire cellular network, schizophrenia being a prime example of this (Hunsberger et al., 2009). The wide-ranging effects of miRNA, with their ability to target hundreds of genes (Hunsberger et al., 2009) make it a molecule of great interest in schizophrenia research. miRNA are single stranded, non-coding RNA molecules typically 21-23nt in length (Dinan, 2010). miRNA are able to regulate gene expression by attaching to mRNA strands based on complementarity of sequence. The degree to which there is complementarity will influence whether the mRNA strand targeted is silenced or degraded. Evaluating miRNAs as biomarkers for schizophrenia is a logical next step as they have been shown to be key components of gene regulation in the development of the central nervous system (CNS) (Smrt et al., 2010; Meza-Sosa et al., 2012). Greater than 50% of identified miRNAs are expressed either solely or principally in the brain (Dinan, 2010). Thus, miRNAs could represent a link between the numerous genes implicated in schizophrenia as they are able to target hundreds of genes making it capable of explaining the varied aspects of the disease process (Hunsberger et al., 2009). The dysregulation of miRNA biogenesis leading to abnormal levels of miRNA in those with schizophrenia or schizoaffective disorders is an area providing very promising results. A key protein implicated in this dysregulation is Dgcr8, which is located on chromosome 22, (Shiohama et al., 2003) within a section (22q11.2) known to be deleted in some patients with schizophrenia (Bassett and Chow, 2008). Santarelli et al. (2011) was able to corroborate the up-regulation of Dgcr8 while also demonstrating the higher abundance of Dicer, another molecule involved in miRNA biogenesis in schizophrenia patients as compared to normal controls through utilization of qRT-PCR (Santarelli et al., 2011).

### Postmortem miRNA dysregulation in schizophrenia

Work by (Beveridge et al., 2010) investigating miRNA expression profiles in both the superior temporal gyrus (STG) and dorsolateral dlPFC illustrated an important global increase in miRNA expression in schizophrenia compared to healthy controls. A total of 59 miRNA were demonstrated to be up-regulated with the miR-105 family of miRNA as well as miR-107 most noticeably expressed at higher levels. This miRNA increase is despite normal pri-miRNA transcripts and gene expression, the difference lies in the increased pre-miRNA concentration. Key genes in the biogenesis of miRNA are also up-regulated leading to the described results. Dgcr8 up-regulation can be found in the STG while up-regulation of Dgcr8 as well as Drosha and Dicer1 were demonstrated in the dlPFC (Xu et al., 2008). It has also been demonstrated that miR-195, which is known to suppress colorectal carcinoma tumor development in the periphery (Liu et al., 2010), as well as miR-30a-5p down-regulates the expression of brain derived neurotrophic factor (BDNF) which consequently cascades into a decreased concentration of GABAergic genes, principally NPY and somatostatin (SST), in the dlPFC of schizophrenic patients as compared to unaffected controls (Mellios et al., 2009; Mellios and Sur, 2012). Investigation revealed that miR-346, a miRNA that targets genes associated with schizophrenia, is encoded within the glutamate receptor ionotropic delta 1 (GRID1) gene (Zhu et al., 2009) which itself was shown previously to be a factor in schizophrenia susceptibility (Treutlein et al., 2009). An investigation of Scandinavian populations (Danish, Swedish, and Norwegian) looking at 18 single nucleotide polymorphisms (SNPs) revealed two SNPs (rs17578796 and rs1700) related to miRNAs miR-206 and miR-198, respectively. These miRNAs showed a significant correlation to schizophrenia as compared to the control group in Danish and Norwegian populations. Of the genes targeted by both these miRNAs, eight of the 15 are known to be transcription factors or interaction partners in the network associated with schizophrenia (Hansen et al., 2007). A further 16 miRNAs that have been found to be differentially expressed in the PFC between schizophrenia and normal controls were identified by Perkins et al. (2007), 15 were seen to be expressed at lower levels in those affected and one was up-regulated. Of the 16 miRNAs, 12 were further investigated using qRT-PCR with seven of the original miRNAs being shown to express at significantly lower concentrations. An additional seven miRNAs have been identified as being dysregulated in the PFC of schizophrenia patients after an initial investigation of 667 candidate miRNAs were analysed using a real-time PCR based Taqman Low Density Array (TLDA). Further study of miR-34a, miR-132, and miR-212, from the 7 initially identified, revealed a significant negative correlation between miR-132 and miR-212 with tyrosine hydroxylase (TH) and phosphogluconate dehydrogenase (PGD) (Kim et al., 2010). A genome wide association study (GWAS) investigating 21,856 subjects with European ancestry and a further 29,839 independent subjects revealed seven mi-RNA related SNPs, five of which were new findings (REF). The strongest correlation was with a SNP, rs1625579, found in the transcript of miR-137. miR-137 is found on chromosome 1p22 in a non-protein-coding RNA gene *AK094607* that is known for its importance to neuronal development (Smrt et al., 2010). Of the other SNPs found, an additional four of them were shown to target the same gene (Schizophrenia Psychiatric Genome-Wide Association Study, 2011). Finally, another recent post-mortem investigation miRNAs in prefrontal cortices of patients with schizophrenia and bipolar disorder revealed that two miRNAs, miR-497 and miR-29c were significantly increased in patients with bipolar disorder vs. controls (Banigan et al., 2013). Changes in expression of these two miRNAs may reflect differences between schizophrenia and bipolar disorder, however, given that this investigation failed to replicate previous miRNA findings for the dlPFC brings into question the consistency in miRNA dysregulation signatures across cohorts. As the miRNA field progresses attention to treatment related changes in these markers will prove dividend, with a recent study demonstrating that some implicated miRNAs for neuropsychiatric disease like, miR-34a above are regulated significantly by the mood stabilizers lithium and sodium valproate (Hunsberger et al., 2013).

### Blood based dysregulation of miRNAs in schizophrenia

Recently, a blood-based miRNA study using PBMCs reported the significant down-regulation of 83 miRNA among individuals with schizophrenia compared to controls. Of those 83 miRNA showing down-regulation, 20% were transcribed from a single maternally expressed, imprinted locus, DLK1-DIO3 (Beveridge and Cairns, 2012). Another study employed mononuclear leukocytes as the cells of interest and completed a genome wide miRNA expression profile of 365 miRNA. An miRNA signature comprising seven miRNA for schizophrenia arose, six up-regulated and one down-regulated. Although these initial results are promising further work is required to determine the if these observed effects are a results of antipsychotic, alcohol, and/or illicit drug exposure rather than underling pathophysiology of schizophrenia (Lai et al., 2011). In relation to biomarker investigations in other living tissues, a recent study has demonstrated that miR-382 was elevated in the olfactory epithelium taken from patients with schizophrenia (Mor et al., 2013), this is of significance as miR-382 regulates expression of fibroblast growth factor receptor-1 (*FGFR1)* that has been previously linked to schizophrenia (Jungerius et al., 2008).

## Conclusion

Despite some overlap between structural, cellular, and molecular data generated from post-mortem investigations, functional neuroimaging studies and blood-based biomarker investigations, the full contribution and impact of past post-mortem literature on biomarker discovery remains to be unearthed. This is largely due to advantages and disadvantages in these different modalities to assess alterations in candidate gene and proteins. For post-mortem investigations, they have the distinct advantage of being able to assess the macro- and microstructure of the brain in a very detailed way in order to map pathways that might be affected in schizophrenia. To date this same resolution cannot be achieved for neuroimaging studies, although the field is technologically evolving rapidly. Given the growing evidence for glutamatergic dysregulation as being a potential driver of schizophrenia and psychosis related pathways, an increase in the number of functional neuroimaging studies to investigate glutamatergic receptor populations is likely. A significant disadvantage of post-mortem investigations is that they are confounded by a number of artifacts, including fixation times, post-mortem interval, and alterations in pH, not to mention trying to account for medication effects. Controlling for these variables in post-mortem studies is challenging, however, with well-characterized brain cohorts this can be achieved. Nevertheless, differences in these variables that can influence both gene and protein expression may account for some discrepancies between post-mortem and functional neuroimaging studies. With regards to blood based biomarker research utilizing high throughput technologies such as microarrays for genotyping and gene expression are relatively new fields, and therefore time is required for the full impact of such investigations to providing biomarkers for diagnosis, prognosis and intervention. While many future biomarker investigations for schizophrenia and psychosis may still largely be hypothesis-free, the growing literature and relative consistency in the data implicating genes such as *NRG1* and the glutamatergic system as a whole are providing researchers within neuropsychiatric genetics with more mechanistic correlates of these biomarkers. In this way, post-mortem literature can act to drive blood-based biomarker investigations by looking for correlates of gene and protein dysfunction within the blood and also via functional changes using neuroimaging. Although blood-based biomarker investigations are exciting and have a realistic potential in uncovering candidate genes and/or proteins relating to schizophrenia, care must be taken in the interpretation of initial preliminary findings to prevent generation of spurious paths of research. By implementing careful study design many of these potential confounders can be reduced or accounted for. In this regard, problems exist in standardization of diagnosis, collection and processing of samples, platform design, and evolution in consistency, laboratory techniques, and tools used for analysis (Chow et al., 2012) Other important potential confounding variables relating to sampling of mixed proportions of PBMCs, and differences in gene expression that exist between these (Palmer et al., 2006) as well as treatment related effects need to be addressed. Investigation of gene expression changes within separate PBMC subpopulations and treatment related gene expression via *in vitro* exposure to various antipsychotic medications may provide one way to address these concerns. Nonetheless as the field of biomarker discovery evolves, links with previous post-mortem data and also current investigations will be made and will potentially add a level of strength to the validity of such candidates. This will be aided technologically by more sophisticated and accurate ways of measuring gene expression such as RNAseq and more sensitive and stringent validation approached such as digital PCR. A point to bear in mind, and that was already mentioned within the introduction is that biomarkers need not necessarily reflect directly brain related changes in patients with psychosis but instead may be purely associative and related to symptoms without a clear pathogenic mechanism of involvement. The strength of these biomarkers lies within the predictive capability in deducing susceptibility, diagnosis or potentially as prognostic indicators, and while a link with brain related changes may exist and be preferential it may not be evidenced straight away. Establishing this link between a biomarker and neuropathophysiology will be one of the ways that future post-mortem investigations will contribute to resolving biomarkers for schizophrenia and psychosis. This approach will be bolstered by investigations of factors such as miRNAs in both blood and brain compartments and the corroboration of these regulatory elements.

### Conflict of interest statement

The authors declare that the research was conducted in the absence of any commercial or financial relationships that could be construed as a potential conflict of interest.
